# Maternal Protein Restriction in Two Successive Generations Impairs Mitochondrial Electron Coupling in the Progeny’s Brainstem of *Wistar* Rats From Both Sexes

**DOI:** 10.3389/fnins.2019.00203

**Published:** 2019-03-14

**Authors:** David F. Santana, Diorginis S. Ferreira, Glauber Ruda F. Braz, Shirley M. S. Sousa, Tercya Lucidi de Araújo Silva, Dayane Aparecida Gomes, Mariana P. Fernandes, Belmira Lara Andrade-da-Costa, Claudia J. Lagranha

**Affiliations:** ^1^Graduate Program in Neuroscience and Behaviour, Universidade Federal de Pernambuco, Recife, Brazil; ^2^Colegiado de Educação Física, Federal University of São Francisco Valley, Petrolina, Brazil; ^3^Departamento de Fisiologia e Farmacologia, Centro de Ciências Biológicas, Universidade Federal de Pernambuco, Recife, Brazil; ^4^Graduate Program in Nutrition, Physical Activity and Phenotypic Plasticity, Academic Center of Vitoria – Universidade Federal de Pernambuco, Vitória de Santo Antão, Brazil; ^5^Núcleo de Educação Física e Ciências do Esporte, Centro Acadêmico de Vitória, Universidade Federal de Pernambuco, Recife, Brazil

**Keywords:** mitochondria, intergenerational, low-protein diet, brainstem, gender, rats

## Abstract

Maternal protein deficiency during the critical development period of the progeny disturbs mitochondrial metabolism in the brainstem, which increases the risk of developing cardiovascular diseases in the first-generation (F1) offspring, but is unknown if this effect persists in the second-generation (F2) offspring. The study tested whether mitochondrial health and oxidative balance will be restored in F2 rats. Male and female rats were divided into six groups according to the diet fed to their mothers throughout gestation and lactation periods. These groups were: (1) normoprotein (NP) and (2) low-protein (LP) rats of the first filial generation (F1-NP and F1-LP, respectively) and (3) NP and (4) LP rats of the second filial generation (F2-NP and F2-LP, respectively). After weaning, all groups received commercial chow and a portion of each group was sacrificed on the 30th day of life for determination of mitochondrial and oxidative parameters. The remaining portion of the F1 group was mated at adulthood and fed an NP or LP diet during the periods of gestation and lactation, to produce progeny belonging to (5) F2R-NP and (6) F2R-LP group, respectively. Our results demonstrated that male F1-LP rats suffered mitochondrial impairment associated with an 89% higher production of reactive species (RS) and 137% higher oxidative stress biomarkers, but that the oxidative stress was blunted in female F1-LP animals despite the antioxidant impairment. In the second generation following F0 malnutrition, brainstem antioxidant defenses were restored in the F2-LP group of both sexes. However, F2R-LP offspring, exposed to LP in the diets of the two preceding generations displayed a RS overproduction with a concomitant decrease in mitochondrial bioenergetics. Our findings demonstrate that nutritional stress during the reproductive life of the mother can negatively affect mitochondrial metabolism and oxidative balance in the brainstem of F1 progeny, but that restoration of a normal diet during the reproductive life of those individuals leads toward a mitochondrial recovery in their own (F2) progeny. Otherwise, if protein deprivation is continued from the F0 generation and into the F1 generation, the F2 progeny will exhibit no recovery, but instead will remain vulnerable to further oxidative damage.

## Introduction

The Developmental Origins of Health and Disease Hypothesis posits that environmental stimuli encountered during critical periods of development, including embryonic, fetal and neonatal life, can induce long-lasting changes in the morphology and physiology of the fully developed individual (Bateson et al., 2004; West-Eberhard, 2005). Among the mechanisms proposed for the developmental origin of adult disease are unbalanced levels of micro- or macronutrients that cause oxidative stress-related damage to critical biomolecular compounds (Luo et al., 2006; Martin-Gronert and Ozanne, 2012; Rashid et al., 2018) which, in turn, enter into signaling pathways underlying several chronic degenerative diseases, including cardiovascular diseases (CVD) (Mansego et al., 2011; Sheeran and Pepe, 2017).

According to the World Health Organization (WHO), nearly 17.5 million deaths per year around the world are associated with CVD, 9.4 million of which have hypertension as the main cause (WHO, 2014). Although, complex and multifactorial etiologies underlie the occurrence of chronic hypertension (Hering et al., 2017), it is suspected that approximately 50% of all cases of hypertension have a neurogenic origin, wherein sympathetic over-activation of central pathways of blood pressure regulation has been described (Guyenet, 2006; Esler et al., 2010). In addition, several studies have demonstrated that this neurogenic hypertension can be triggered by mitochondrial impairment and oxidative stress into the brainstem (Chan et al., 2006, 2009b; Hirooka, 2008; Lopez-Campistrous et al., 2008; Dampney, 2016).

Several reports showed that environmental insult during critical periods of development can induce oxidative imbalance in various components of the central nervous system (CNS) of the adult (Bonatto et al., 2005, 2006; Feoli et al., 2006; Ferreira et al., 2016c). Moreover, our laboratory has demonstrated that a low-protein maternal diet during the perinatal period of her offspring results in impaired mitochondrial function and antioxidant capacity throughout the lives of those progeny, and an increased risk of developing CVD in adulthood (Ferreira et al., 2015, 2016a, 2018; de Brito Alves et al., 2016; de Sousa et al., 2017). There is also evidence that the increased risk of CVD may not affect only the immediate offspring of an undernourished parent, but, it that might also be transmitted across successive generations (i.e., children, grandchildren, great-grandchildren) in several possible ways, including non-Mendelian inheritance via maternal mitochondria (Zambrano et al., 2005; Zambrano, 2009; Dunn and Bale, 2011; Bale, 2015; Aiken et al., 2016; Saben et al., 2016). This means of transmitting parental damage to succeeding generations is particularly relevant to nutritional deficits during development (Woods et al., 2018), as a number of experimental studies have demonstrated long-lasting changes in mitochondrial functions such as apoptosis, calcium control, redox homeostasis and energy supply (Bertram et al., 2008; Ponzio et al., 2012; Yin and Cadenas, 2015; Saben et al., 2016) following maternal malnutrition.

On the other hand, several studies have described the enhancement of cell-protective systems following repeated environmental insult, including the up-regulation of various antioxidant mechanisms that lend resilience to oxidative stress and the amelioration of oxidative damage in the progeny of malnourished mothers (Pickering et al., 2013; Banos-Gomez et al., 2017).

Among the factors contributing to the degree of oxidative damage, and conversely the upregulation of antioxidant defenses in individuals exposed to malnutrition during gestation/lactation, is the gender, which greatly influences the relative amount of estrogen present both during the period of mitochondrial damage in the mother, and later in life when damage first becomes evident in the progeny (Lagranha et al., 2010; Braz et al., 2017; Silva et al., 2018). Clearly then, a maternal low-protein diet would be expected to affect male and female offspring differently in the F1 generation, and perhaps into succeeding generations.

We have previously shown that maternal protein deficiency during a critical period of rat brain development results in mitochondrial dysfunction in the brainstem of her offspring throughout life (Ferreira et al., 2016a, 2018; de Sousa et al., 2017). In the current study, we investigate whether a maternal low-protein diet differentially affects mitochondrial bioenergetics and oxidative balance in the male and female brainstem not only in the F1 generation, but also in the generation that follows. In addition, we examine whether oxidative brainstem resilience through upregulation of protective anti-oxidant mechanisms occurs in the second filial (F2) generation when that generation is re-exposed to a maternal low-protein diet.

## Materials and Methods

### Ethics Statement

This study was carried out in accordance with the recommendations of the National Institutes of Health guide for animal experimentation (NIH Publications N^°^. 80-23, revised 1978). The protocol was approved by the Ethical Committee of the Bioscience Center of the Federal University of Pernambuco (n° 23076.018417/2013-73).

### Experimental Groups and Diet

#### F1 Offspring

**FIGURE 1:**
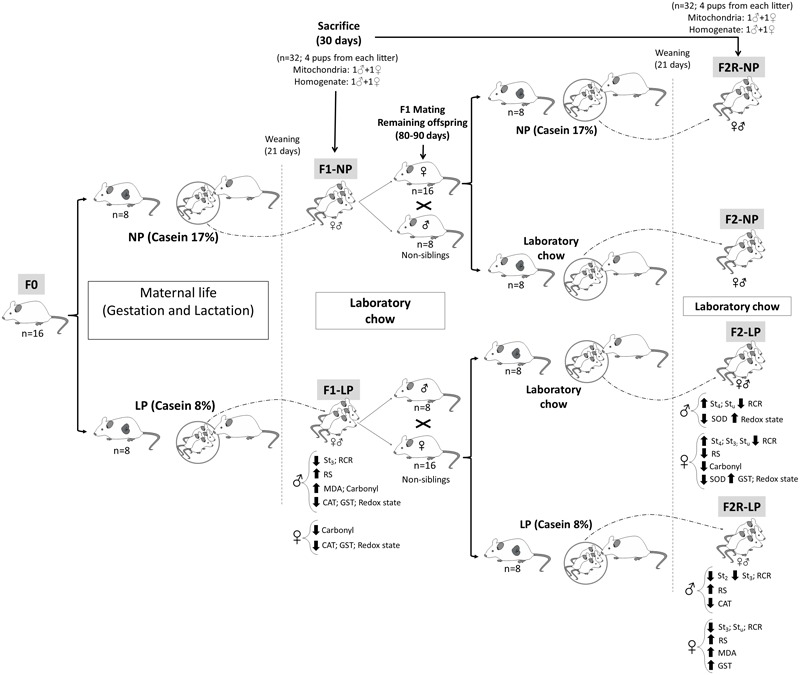
Diagram of the experimental design. Normal protein diet (NP); Low-protein diet (LP); Female rats before mating (F0); First generation offspring of mothers that were fed either normal protein (F1-NP) or low-protein (F1-LP) diets; Offspring of F0 grandmothers that were fed normal protein (F2-NP) or low-protein (F2-LP) diets; Offspring of two successive female generations (grandmothers and mothers) that were fed normal protein (FR2-NP) or low protein (FR2-LP) diets; Basal consumption of O_2_ by the mitochondria (St_2_); Mitochondrial consumption of O_2_ when stimulated with ADP (St_3_); Mitochondrial consumption of O_2_ following ATP synthase inhibition (St_4_); O_2_ consumption by an uncoupled mitochondria (St_u_); Respiratory Control Ratio (RCR); Reactive species (RS); Biomarker of oxidative damage to lipids (MDA); The enzymatic activities of- Superoxide dismutase (SOD); Catalase (CAT); and Glutathione-S-transferase (GST).

Nulliparous Wistar rats at 90 days of age and weighing 250–270 g were mated in a ratio of two females to one male, and pregnancy was determined by the presence of spermatozoa in vaginal smears. The pregnant rats (*n* = 16) were divided into two groups according to the protein content of their respective diets, which were formulated in the Laboratory of Dietetic Techniques at the Federal University of Pernambuco, as previously described (Reeves et al., 1993; Ferreira et al., 2016a). These diets were: (1) 17% protein (*n* = 8; normo-protein, NP) or (2) 8% protein (*n* = 8; low-protein, LP). Both diets were isocaloric and had casein as the only source of protein, with other dietary components added based on recommendations of the American Institute of Nutrition (AIN). NP dams delivered 12–15 pups, while LP delivered 8–11. However, no difference in sex ratio between groups was observed. Twenty-four hours after birth, the litters were standardized to eight pups per litter in the first generation (F1). The litters were maintained on their respective NP and LP diets during a lactation period lasting 21 days, then received laboratory chow (Labina; Purina Agriband, Brazil) until they reached an age of 30 days of life, at which time a part of each group was sacrificed by decapitation for the experimental procedures. Two males and two females were sacrificed in each litter, and experimental analyses were performed with 1 rat from each litter (i.e., 1 male and 1 female for the mitochondria assays, as well as for the total homogenate) (see diagram in Figure 1).

#### F2 Offspring

Males (*n* = 16) and females (*n* = 32) in the F1 generation that were not sacrificed were maintained on standard laboratory chow until reaching 80–90 days of age, at which time they were mated to produce the F2 generation of offspring. Mating was always performed between rats from different litters, in a ratio of two females to one male. Once pregnancy was verified, the pregnant F1 rats were assigned to receive either (1) the diet that their mothers’ had received, or (2) a diet of laboratory chow. Thus, the following groups were formed: (1) F2-NP (*n* = 8): pups descended from grandmothers that received a normoprotein diet and mothers that received laboratory chow during gestation and lactation; (2) F2-LP (*n* = 8): pups descended from grandmothers that received a low-protein diet and mothers that received laboratory chow during gestation and lactation; (3) F2R-NP (*n* = 8): pups descended from grandmothers and mothers that received normoprotein diet during gestation and lactation; and (4) F2R-LP (*n* = 8): pups descended from grandmothers and mothers that received low-protein diet during gestation and lactation. No difference in gender ratio or other litter characteristics was observed between the F1 and F2 animals. All groups were maintained on their respective diets until weaning (21 days of age), at which time they received laboratory chow (Labina; Purina Agriband) until, being sacrificed for experimental analyses at 30 days of age.

### Preparation of Brainstem Mitochondria

After the brainstems were removed, they were immediately minced and homogenized in an ice-cold mitochondrial isolation buffer containing 225 mM mannitol, 75 mM sucrose, 4 mM HEPES, 2 mM taurine and 0.5 mM EGTA, pH 7.4, using a potter-Elvehjem pestle and glass tube connected to a homogenizer (IKA^@^ RW20 digital). Homogenates were centrifuged for 5 min at 1,180 × *g* maintained at 4°C and the resulting supernatants were centrifuged for an additional 10 min at 12,470 × *g* and 4°C. After the last centrifugation, the pellets containing isolated mitochondria were re-suspended in respiration buffer (RB) consisting of 120 mM KCl, 4 mM HEPES, 5 mM K_2_HPO_4_ and 0.2% BSA (w/v), pH 7.4. Mitochondria were kept on ice until assay (Lagranha et al., 2010; Ferreira et al., 2016a).

#### Measurement of Mitochondrial Respiration

Mitochondrial respiration is a cogent measure of the ability of the organelle to produce ATP in response to different energy demands. The assay is based on the mitochondrial capacity to consume O_2_ in response to the electron coupling-related proton current (Chance and Williams, 1955; Brand and Nicholls, 2011). Here, we used a 600 SL chamber connected to a Clark-type oxygen electrode (Hansatech Instruments, Pentney King’s Lynn, United Kingdom) at 28°C to monitor the O_2_ consumption under different conditions (Ferreira et al., 2016a). Initially, mitochondria were incubated in RB (1 mg protein/mL) containing only Complex I substrates (10 mM glutamate and 0.4 mM malate) (basal state/state 2). This state restricts the mitochondria to a minimal consumption of O_2_ due to the scarcity of ADP in the assay. Thereafter, ATP synthesis was stimulated by adding 0.5 mM ADP (state 3/ADP-stimulated), at which time, mitochondria use O_2_ as the final electron acceptor while phosphorylating ADP to ATP via ATP synthase activation. State 3 was ended by the addition of 1.2 μM oligomycin (resting state) to decrease O_2_ consumption through inhibition of ATP synthase activity. The last state evaluated was the uncoupled state, measured by adding 5 μM carbonyl cyanide *m*-chlorophenyl hydrazone (CCCP), a protonophore that increases the velocity of O_2_ consumption by uncoupling the mitochondria (Goldsby and Heytler, 1963). In addition, ADP-stimulated/resting states were used to assess the mitochondrial respiratory control ratio (RCR), a classic indicator of mitochondrial “health” (Nicholls and Bernson, 1977; Brand and Nicholls, 2011).

#### Measurement of Mitochondrial Reactive Species (RS) Production

RS production was assessed by the dihydrodichlorofluorescein diacetate (H_2_DCF-DA) method (Ferreira et al., 2016a). Briefly, 0.1 mg of mitochondria were incubated in RB with complex I substrates, as described in Section “Measurement of Mitochondrial Respiration,” followed by the addition of 5 μM (H_2_DCF-DA), which in the presence of reactive species form a product that fluoresces at 485 nm excitation and 530 nm emission. The reaction was followed by gentle shaking for 8 min in a FLUOstar OMEGA spectrophotometer (BMG Labtech, United States) at 28°C. The results were expressed as a percentage of H_2_DCF-DA fluorescence yielded.

### Sample Preparation for Oxidative Stress and Antioxidant Analyses in the Total Homogenate

Frozen brainstems were immersed in a cold buffer containing 50 mM TRIS and 1 mM EDTA, pH 7.4, with the addition of 1 mM sodium orthovanadate and 200 μg/mL phenylmethanesulfonylfluoride. Samples were then homogenized with an IKA^@^ RW20 digital homogenizer using a potter-Elvehjem pestle in a glass tube on ice. Homogenates were centrifuged at 1,180 × *g* for 10 min at 4°C. Protein concentration of the supernatants was determined by the Bradford method which measures absorption at 595 nm at RT (Bradford, 1976).

#### Evaluation of Lipid Peroxidation (MDA Levels)

Lipid peroxidation was analyzed using malondialdehyde (MDA) levels, as previously published (Buege and Aust, 1978). Three hundred μg protein was sequentially mixed to in 30% (w/v) trichloroacetic acid (TCA) and 10 mM TRIS buffer at 30°C, pH 7.4. This mixture was centrifuged at 2,500 × *g* for 10 min, and the supernatant was boiled for 15 min with 0.73% (w/v) thiobarbituric acid. The resulting pink pigment was then measured at 535 nm absorption at RT and the extinction coefficient used to determine the MDA level.

#### Determination of Protein Oxidation (Carbonyl Content)

Protein oxidation was assessed using the procedures outlined by Reznick and Packer (1994). Briefly, the samples were placed on ice and 30% (w/v) TCA was added, and the mix was then centrifuged for 14 min at 1,180 × *g*. The pellet was re-suspended in 10 mM 2,4 dinitrophenylhydrazine and immediately incubated in a dark-room for 1 h with agitation every 15 min. Samples were washed and centrifuged three times in ethyl/acetate buffer, and the final pellet was re-suspended in 6 M guanidine hydrochloride, incubated for 30 min at 37°C and the absorbance read at 370 nm. Results were expressed as a percentage of the control group value.

### Measurement of Total Superoxide Dismutase (SOD) Activity

Total SOD activity was evaluated following the protocol developed by Misra and Fridovich (1972). In brief, 300 μg of protein was added to 50 mM carbonate buffer with 0.1 mM EDTA, pH 10.2, and the reaction was started with the addition of 150 mM epinephrine. SOD activity was determined by measuring the inhibition epinephrine auto-oxidation at 30°C for 1.5 min as indicated by the decrease in absorbance at 480 nm. Results expressed as a percentage of the control group value.

#### Measurement of Catalase (CAT) Activity

The assay for CAT activity was performed as previously described by Aebi (1984). Briefly, 0.3 M hydrogen peroxide and 300 μg protein were added to a 50 mM phosphate buffer, pH 7.0 at 20°C and absorption decay in the mix was monitored for 3 min at 240 nm to indicate CAT activity. Results were expressed as a percentage of the control group value.

#### Measurement of Glutathione-S-Transferase (GST) Activity

GST activity was measured as described previously by Habig and Jakoby (1981). Three hundred μg of protein was incubated in a 0.1 M phosphate buffer, pH 6.5 containing 1 mM EDTA at 30°C. After the addition of 1 mM 1 chloro-2.4-dinitrobenzene and 1 mM reduced glutathione (GSH), the formation of 2.4-dinitrophenyl-S-glutathione was followed for 1 min at 340 nm. Results were expressed as a percentage of the control group value.

#### Measurement of Reduced Glutathione (GSH) and Oxidized Glutathione (GSSG)

To assess GSH levels, the samples were diluted (1:10) in a 0.1 M phosphate buffer containing 5 mM EDTA, pH 8.0, and an aliquot from the diluted sample was incubated with *o*-Phthaldialdehyde (OPT) at RT for 15 min. After incubation, the fluorescence intensity of the mix was measured at 420 nm emission and 350 nm excitation and then GSH in each sample was determined by comparison to a standard curve of known GSH concentrations. To determine GSSG levels, the samples were incubated with 0.04 M *N*-ethylmaleimide for 30 min at RT followed by addition of 0.1M NaOH, and after incubation, fluorescence measurements and calculations of GSSG levels were performed as described in the GSH assay above. The results for GSH and/or GSSG levels were expressed in units of μmol/mg protein. Redox state was determined by the ratio of GSH/GSSG as previously described (Hissin and Hilf, 1976).

### Statistical Analyses

All values are expressed as mean ± SEM. Once the data were tested for normal distribution (Kolmogorov–Smirnov), either the unpaired Student’s *t*-test or the Mann–Whitney test (depending on normality) was employed to assess the significance of differences between groups. Comparisons were considered statistically significant at *p* ≤ 0.05. All statistical analyses were performed using GraphPad Prism 6.0^®^ software (GraphPad Software, Inc.).

## Results

### First Generation (F1)

It has been suggested that the females are more protected from oxidative damage than males. As a first step in this investigation, we addressed a possible gender influence on oxidative parameters in F1 rats born and nursed by normoprotein mothers. Our findings corroborate the existing literature on this subject by demonstrating that female F1 offspring from the normoprotein group have lower levels of oxidative biomarkers (i.e., oxidized lipid and protein), and enhanced antioxidant capacity (CAT activity and Redox status) in the brainstem compared with male offspring in the same cohort (Supplementary Data). After determining a protective effect of female gender on oxidative parameters in F1 animals, we addressed the effects of maternal low-protein diet in the brainstem of male and female in the subsequent (F2) generation.

**FIGURE 2:**
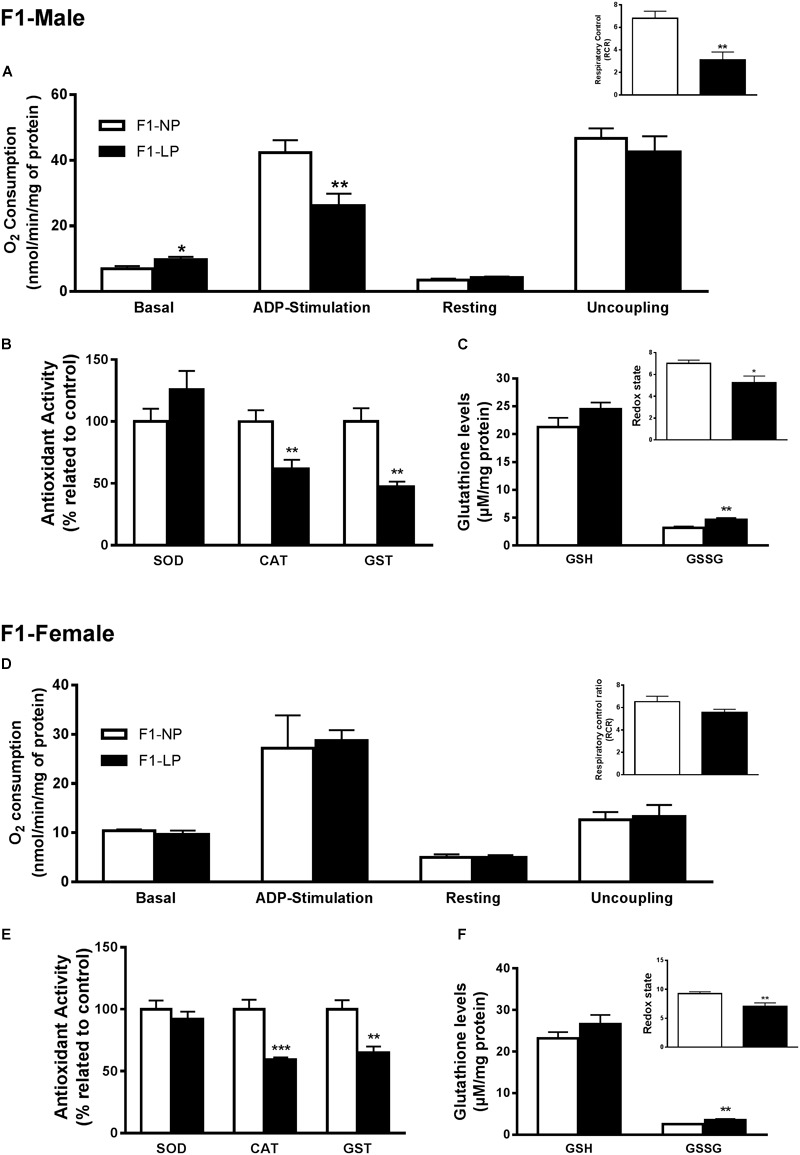
Effects of maternal protein restriction in the brainstem metabolism of first generation (F1) progeny. Male offspring of mothers that were fed either normal protein or low protein diets (F1-NP vs. F1-LP, respectively): mitochondrial O_2_ consumption and respiratory control ratio **(A)**; enzymatic antioxidant capacity **(B)**; non-enzymatic antioxidant system and redox state **(C)**. Data are presented as mean ± SEM. ^∗^*p* ≤ 0.05. ^∗∗^*p* ≤ 0.01; ^∗∗∗^*p* ≤ 0.001 by unpaired Student’s *t*-test (*n* = 4–8). Female offspring of normal protein vs. low protein mothers (F1-NP vs. F1-LP, respectively): mitochondrial O_2_ consumption and respiratory control ratio **(D)**; enzymatic antioxidant capacity **(E)**; non-enzymatic antioxidant system and redox state **(F)**. Data are presented as mean ± SEM. ^∗^*p* ≤ 0.05. ^∗∗^*p* ≤ 0.01; ^∗∗∗^*p* ≤ 0.001 using an unpaired Student’s *t*-test (*n* = 4–8).

In male offspring of the first generation, the basal state of the LP group showed higher O_2_ consumption than the NP group (F1-NP: 6.95 ± 0.81 vs. F1-LP: 9.65 ± 0.89 nmol O_2_/mL; *p* = 0.0497; *n* = 6–7) (Figure 2A). However, when stimulated with ADP, the LP animals were unable to increase their O_2_ consumption as the NP animals did, culminating in a lower state 3 for the LP group (F1-NP: 42.40 ± 3.76 vs. F1-LP: 26.18 ± 3.62 nmol O_2_/mL; *p* = 0.013; *n* = 5-6) (Figure 2A). Although no further differences were found across the coupling states, the reduced responsiveness to ADP in the LP offspring lowered the RCR (F1-NP: 6.81 ± 0.62 vs. F1-LP: 3.13 ± 0.68; *p* = 0.004; *n* = 5) (Figure 2A). Additionally, the F1-LP male offspring produced more reactive species than the F1-NP group (F1-NP: 100.0 ± 10.21 vs. F1-LP: 189.8 ± 22.48 percentage compared to the control; *p* = 0.0066; *n* = 5) (Table 1). As result, the male offspring exhibited increased oxidative damage to both lipids and proteins (Lipids - F1-NP: 100.0 ± 4.32 vs. F1-LP: 146.9 ± 7.61 percent MDA compared to the control; *p* = 0.0007; *n* = 5; proteins - F1-NP: 100.0 ± 9.72 vs. F1-LP: 190.6 ± 33.42 percent carbonyl compared to the control; *p* = 0.0264; *n* = 6) (Table 1). The augmented oxidative stress is consistent with the significant decreases in both enzymatic (Figure 2B) and non-enzymatic (Figure 2C) antioxidant systems (*n* = 5–6) observed in male F1 offspring with an LP background compared to those with an NP background.

**Table 1:** Production of reactive species (RS) and oxidative biomarkers in the brainstem of male and female progeny of F0 mothers that were protein restricted during gestation and the lactation.

		Male		Female	
		NP	LP	NP	LP
F1	RS	100.0 ± 10.21	189.8 ± 22.48^∗∗^	100.0 ± 7.86	75.64 ± 13.63
	MDA	100.0 ± 4.32	146.86 ± 7.61^∗∗∗^	100.0 ± 9.40	113.26 ± 8.51
	Carbonyl	100.0 ± 9.72	190.60 ± 33.42^∗^	100.0 ± 5.09	76.05 ± 1.99^∗∗^
F2	RS	100.0 ± 8.17	93.62 ± 8.32	100.0 ± 5.26	77.49 ± 7.26^∗^
	MDA	100.0 ± 6.56	86.85 ± 15.94	100.0 ± 4.62	90.04 ± 3.16
	Carbonyl	100.0 ± 2.42	88.47 ± 13.25	100.0 ± 9.06	72.10 ± 3.26^∗^
F2R	RS	100.0 ± 14.05	285.5 ± 22.93^∗∗^	100.0 ± 6.64	239.9 ± 37.42^∗∗^
	MDA	100.0 ± 12.5	106.63 ± 15.47	100.0 ± 13.83	173.78 ± 16.21^∗∗^
	Carbonyl	100.0 ± 4.20	98.87 ± 5.72	100.0 ± 9.15	73.38 ± 9.54

*F1, first generation progeny; F2, second generation progeny; F2R, second generation progeny re-exposed to nutritional insult during development by protein restriction of their F1 mothers; RS, reactive species; MDA, lipid peroxidation. Data are expressed as a percentage of the control group ± SEM; *n* = 4–6. ^∗^*p* ≤ 0.05, ^∗∗^*p* ≤ 0.01, and ^∗∗∗^*p* ≤ 0.001.*

In marked contrast to the males, female offspring of the first generation showed no differences in either mitochondrial coupling states (Figure 2F) or the production of RS (Figure 2D) between NP and LP individuals. Interestingly, however, the F1-LP female offspring showed increased protection from oxidative damage to protein (F1-NP: 100.0 ± 5.09 vs. F1-LP: 76.06 ± 1.99 percentage carbonyl compared to the control; *p* = 0.0024; *n* = 5) (Table 1) even though their antioxidant capacity was reduced (*n* = 5–6) (Figure 2E,F).

### Second Generation

**FIGURE 3:**
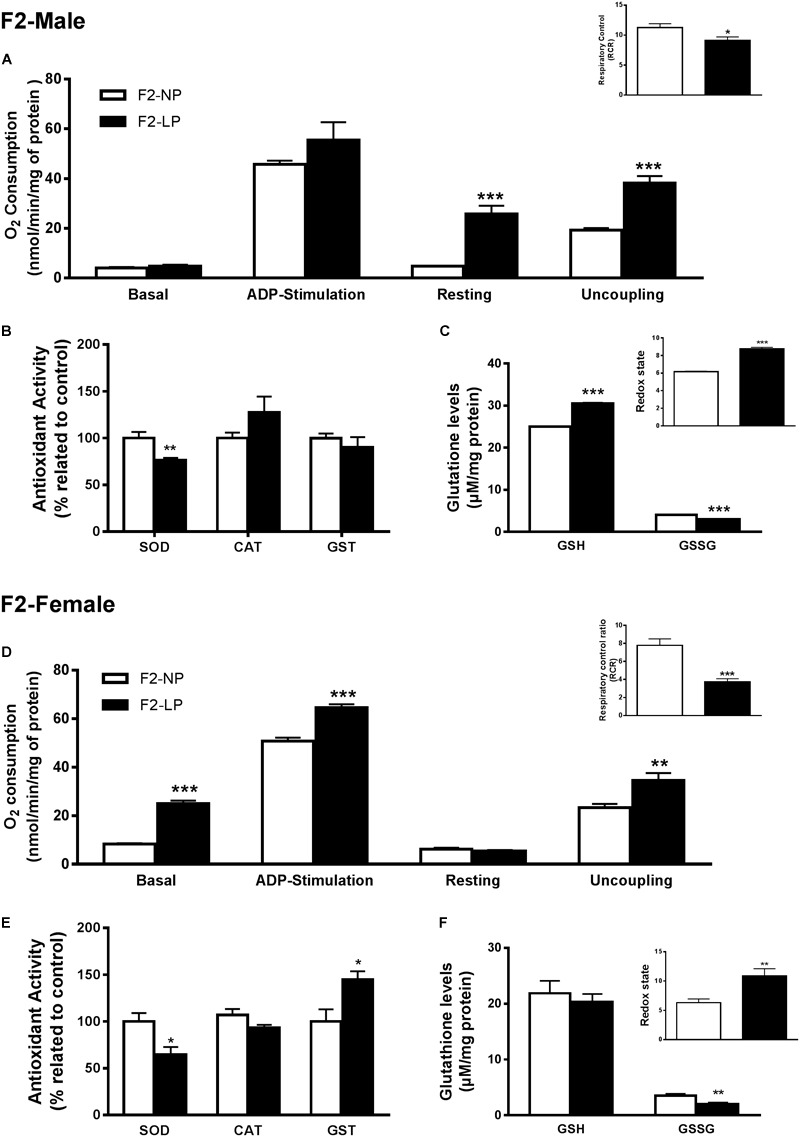
Effects of maternal protein restriction in the brainstem metabolism of the second generation (F2) progeny. Male offspring of F0 grandmothers that were fed normal protein vs. low protein diets (F2-NP vs. F2-LP, respectively): mitochondrial O_2_ consumption and respiratory control ratio **(A)**; enzymatic antioxidant capacity **(B)**; non-enzymatic antioxidant system and redox state **(C)**. Data are presented as mean ± SEM. ^∗^*p* ≤ 0.05. ^∗∗^*p* ≤ 0.01; ^∗∗∗^*p* ≤ 0.001 using an unpaired Student’s *t*-test (*n* = 4–8). Female offspring of F0 grandmothers that were fed normal protein vs. low protein diets (F2-NP vs. F2-LP, respectively): mitochondrial O_2_ consumption and respiratory control ratio **(D)**; enzymatic antioxidant capacity **(E)**; non-enzymatic antioxidant system and redox state **(F)**. Data are presented as mean ± SEM. ^∗^*p* ≤ 0.05. ^∗∗^*p* ≤ 0.01; ^∗∗∗^*p* ≤ 0.00l using an unpaired Student’s *t*-test (*n* = 4–8).

Our findings for the F2 groups showed that impairments in mitochondrial bioenergetics induced by a maternal low-protein diet were extended to the second generation while the non-enzymatic capacity increased, possibly as a compensatory mechanism for the oxidative damage. In males of the F2 generation, no differences were found in either the basal or ADP-stimulated states, although both the resting and uncoupled states were increased in F2-LP offspring compared to NP offspring (F2-NP: 4.74 ± 0.15 vs. F2-LP: 25.8 ± 3.30 nmol O_2_/mL; *p* < 0.0001) and (F2-NP: 19.29 ± 0.84 vs. F2-LP: 38.26 ± 2.78 nmol O_2_/mL; *p* = 0.0002), respectively (*n* = 5–7) (Figure 3A), lowering the RCR in the LP animals (F2-NP: 11.26 ± 0.64 vs. F2-LP: 9.13 ± 0.57; *p* = 0.0294; *n* = 7) (Figure 3A). In regard to oxidative balance, our data demonstrate similar RS production as well as oxidative biomarkers in the F2-NP and F2-LP groups (*n* = 5) (Table 1). On the other hand, the F2-LP group showed a lower capacity to deal with superoxide (*n* = 6) (Figure 3B) that might be offset by the increase in the redox status (*n* = 4) (Figure 3C).

Female LP offspring, despite showing a higher phosphorylation capacity in the ADP-stimulated state (F2-NP: 50.80 ± 1.42 vs. F2-LP: 64.55 ± 6.9 nmol O_2_/mL; *p* = 0.0005), and higher basal and uncoupling states (*n* = 4–6) had a decreased respiratory control ratio, which represents an overall decline in mitochondrial capacity (F2-NP: 7.77 ± 0.72 vs. F2-LP: 3.75 ± 0.33; *p* = 0.0001; *n* = 5) (Figure 3D). Regarding oxidative balance, females of the F2 generation down-regulated their RS production (*n* = 5–6) (Table 1), thereby protecting proteins from oxidative damage (*n* = 5) (Table 1) by dealing with the general electrophilic compounds (*n* = 4–5) (Figure 3E,F).

### Second Generation Re-exposed to Low Protein or Normoprotein

**FIGURE 4:**
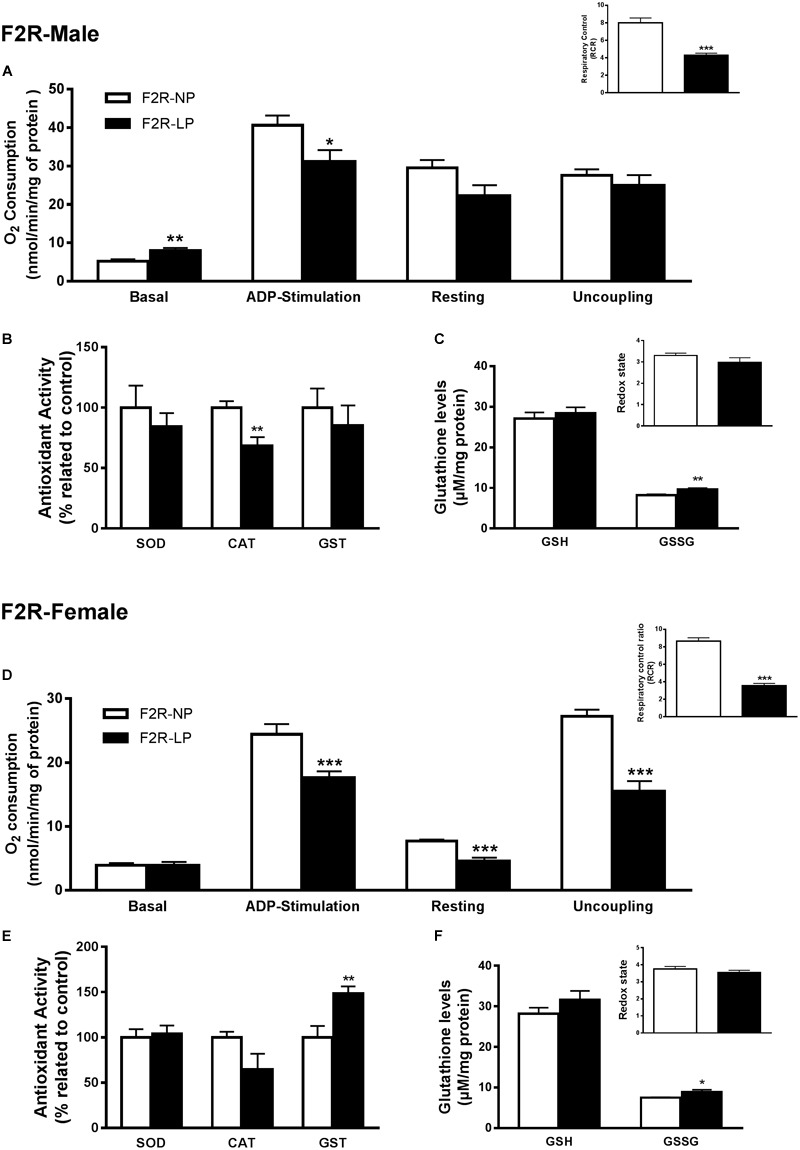
Effects of maternal F0 protein restriction in the brainstem metabolism of second generation (F2) progeny, reinforced by re-imposition of low protein during their gestation and early development. Male offspring of successive female generations (grandmothers and mothers) that were fed normal protein vs. low protein diets (FR2-NP vs. FR2-LP, respectively): mitochondrial O_2_ consumption and respiratory control ratio **(A)**; enzymatic antioxidant capacity **(B)**; Non-enzymatic antioxidant system and redox state **(C)**. Data are presented as mean ± SEM. ^∗^*p* ≤ 0.05. ^∗∗^*p* ≤ 0.01; ^∗∗∗^*p* ≤ 0.001 using an unpaired Student’s *t*-test (*n* = 4–8). Female offspring of successive female generations (grandmothers and mothers) that were fed normal protein vs. low protein diets (FR2-NP vs. FR2-LP, respectively): mitochondrial O_2_ consumption and respiratory control ratio **(D)**; enzymatic antioxidant capacity **(E)**; non-enzymatic antioxidant system and redox state **(F)**. Data presented as mean ± SEM. ^∗^*p* ≤ 0.05. ^∗∗^*p* ≤ 0.01; ^∗∗∗^*p* ≤ 0.001using an unpaired Student’s *t*-test (*n* = 4–8).

Several studies suggest that chronic population-wide conditions in (such as malnutrition) affecting individuals of reproductive age can result in increased susceptibility to those conditions in their F1 offspring and in subsequent generations. To investigate this hypothesis in our model, we evaluated oxidative parameters in the F2 progeny of F1 rats that were exposed to the effects of a low-protein diet during their early development, and then were themselves exposed to the same low protein diet during their own gestation and nursing periods. In male offspring ‘re-exposed’ to a low-protein diet, mitochondria increased their consumption of O_2_ in state 2 (F2R-NP: 5.18 ± 0.51 vs. F2R-LP: 8.011 ± 0.65 nmol O_2_/mL; *p* = 0.007; *n* = 4-5) and decreased the both ADP-stimulated state (F2R-NP: 40.64 ± 2.52 vs. F2R-LP: 31.16 ± 3.0 nmol O_2_/mL; *p* = 0.042; *n* = 4-5) and the RCR (F2R-NP: 8.01 ± 0.53 vs. F2R-LP: 4.27 ± 0.25; *n* = 5) compared to control (Figure 4A). Following re-exposure to LP, RS production increased markedly in LP males (F2R-NP: 100.0 ± 14.05 vs. F2R-LP: 285.5 ± 22.93 percentage compared to the control; *p* < 0.0001; *n* = 5) (Table 1). However, no differences were found in biomarkers of either lipid or protein oxidation (*n* = 4–6) (Table 1) or in antioxidant parameters (*n* = 4–6) (Figure 4B,C).

In contrast to males of the F2 generation, when female offspring were re-exposed to an LP diet, their mitochondria decreased the O_2_ consumption in most of the respiratory states (*n* = 6) (ADP-stimulated, *p* < 0.0001; resting, *p* = 0.0003 and uncoupling, *p* = 0.001), culminating in the decrease of respiratory control ratio and RCR (*p* < 0.0001; *n* = 6) (Figure 4D). As in the males, however, low-protein re-exposure of F2 females promoted RS overproduction (F2R-NP: 100.0 ± 6.64 vs. F2R-LP: 239.9 ± 37.42 percentage compared to the control; *p* = 0.0043; *n* = 4–5) (Table 1) which in the females was accompanied by an increase in lipid damage (F2R-NP: 100.0 ± 13.84 vs. F2R-LP: 173.8 ± 16.21 MDA percentage compared to the control; *p* = 0.0085; *n* = 5) (Table 1), despite the up-regulation of GST activity (*p* = 0.0122; *n* = 4-5) (Figure 4E), and unchanged non-enzymatic defense (Figure 4F).

Information regarding the animal body composition, and additional statistic results can be accessed in the Supplementary Data.

## Discussion

In the present study, we have employed a maternal low-protein diet model to assess the effect of the protein deprivation on the mitochondrial bioenergetics and oxidative balance in the brainstems of progeny born to the protein-deprived mothers, and in the brainstems of rats in the succeeding generation. Furthermore, we evaluated whether the F2 offspring born and nursed by protein-deprived mothers can compensate for oxidative damage when they themselves are ‘re-exposed’ to the same low-protein nutritional insult. Our findings demonstrate that maternal protein restriction disrupts mitochondrial coupling states in all experimental groups except females of the F1 generation. We hypothesize that despite the impairments to mitochondrial function, F2 animals were able to avoid oxidative damage by altering their redox status in response to renewed protein restriction.

Compelling evidence has suggested a relationship between mitochondrial dysfunction and oxidative stress in the brain, and the neuropathogenesis of hypertension (Peterson et al., 2006; Hirooka, 2008; Lopez-Campistrous et al., 2008; Chan and Chan, 2013). Studies from our laboratory have demonstrated that maternal protein restriction during gestation and lactation disrupts mitochondrial bioenergetics, increases RS production, and impairs redox homeostasis in the young and adult brainstem, increasing the risk in those progeny of developing autonomic activity-related hypertension in later life (de Brito Alves et al., 2014; Barros et al., 2015; Ferreira et al., 2015, 2016b; de Brito Alves et al., 2016; de Sousa et al., 2017).

According to Guyenet (2006), impairments in either afferent or efferent autonomic pathways are the underlying cause of neurogenic hypertension, a blood pressure defect characterized by a flaw in autonomic control rather than a dominant renal or vascular irregularity. As previously demonstrated, at 30 days of life, F1-LP male offspring exhibit higher respiratory frequency and ventilation-related chemosensitivity than NP individuals, however, no change in blood pressure was observed (de Brito Alves et al., 2014). Maximo Cardoso et al. (2006) demonstrated that an increase in reactive oxygen species in the fourth ventricle induces pressor responses by unbalancing autonomic control. In spontaneously hypertensive rats, Chan et al. (2006) and Nishihara et al. (2012) demonstrated that the reduction in antioxidant capacity in the rostral ventrolateral medulla (RVLM), a brainstem nucleus involved in the efferent arm of pressure control, contributes to neurogenic hypertension. Further studies from Chan’s laboratory suggested that an impairment in the mitochondrial electron transport chain (ETC), in addition to mitochondrial uncoupling-induced oxidative stress, could contribute to the development of neurogenic hypertension (Chan et al., 2009a,b). Here, we demonstrate that young male F1-LP rats overproduce RS while exhibiting decreased antioxidant capacity. In addition, we observe decreased mitochondrial phosphorylative capacity (low RCR and state 3) that would deprive the brainstem of energy and thus suggest an autonomic imbalance in male offspring.

In isolated mitochondria, energy metabolism relies on three components - substrate oxidation, ATP turnover, and proton leak - which together, control the outward and inward transport of protons across the mitochondrial inner membrane (Hafner et al., 1990). Substrate oxidation involves reactions that prompt the mitochondrial proton motive force (pmf) (substrate uptake, metabolism and electron transport). ATP turnover refers to the reactions that employ the pmf to phosphorylate ADP to ATP and to export it, while proton leak employs the pmf without yielding ATP (Hafner et al., 1990; Brand and Nicholls, 2011). In the brain, Olorunsogo (1989) described how protein restriction affects substrate oxidation by decreasing dehydrogenase activities (e.g., isocitrate and succinate), which in turn could diminish supplies of critical co-factors to the mitochondrial ETC. The authors also stated that the mitochondria of LP animals had decreased their capacity for coupling electron transport, proton pumping, and ATP synthesis, possibly due to the decrease in the cytochrome C oxidase activity in rats with an LP background. Ferreira et al. (2016a) demonstrated that, at 22 days of life, lower phosphorylative capacity in the male F1-LP brainstem is associated with a disruption in mitochondrial membrane potential due to the deceleration of the Krebs cycle and the lower mitochondrial co-factor supply. Such impairments could persist to at least 30 days of life (the age of sacrifice for F1 animals in the present study). However, further studies must be done to address how the maternal LP diet affects mitochondrial components involved in the pmf maintenance in her offspring.

In the present study, we’ve shown that F1-LP mitochondria in the basal state consume more O_2_ than the normal rats, indicating the occurrence of mitochondrial uncoupling (Parker, 1965). Moreover, we’ve shown that the different responsiveness to ADP stimulation is not related to the ATP synthase, since the consumption of O_2_ across the resting and uncoupling states are unaltered. In the adult LP offspring, however, both states following state 3 consumed more O_2_ than in the NP offspring, especially with complex II, suggesting the presence of defects in ATP synthase (Ferreira et al., 2018). In the present study, since, mitochondria appear to respirate equally in the last two states evaluated, we believe as do Olorunsogo (1989), that mitochondria in LP individuals may have lost their capacity for coupling to electron transport, the proton pump and the ATP synthesis. Studies with hypertensive rats have demonstrated similar patterns. Chan et al. (2009b) describe a flaw in the electron coupling across the mitochondrial complexes in the RVLM, while Lopez-Campistrous et al. (2008) point to a dysfunction in brainstem complex I (anti Fe–S protein expression and complex activity).

Regarding mitochondrial bioenergetics in the female offspring, it is recognized that genomic and phenotypic sex differences assure the female a degree of cardioprotection that is unavailable to the males (Lagranha et al., 2010). Rodriguez-Rodriguez et al. (2015) argued that the more favorable global plasma antioxidant status established in malnourished females during perinatal life can contribute to their protection against hypertension in adulthood. In the present study we show that, despite the decrease in antioxidant capacity occurring in the young female F1-LP group, both the mitochondrial bioenergetics and the production of RS were unchanged in face of the nutritional insult.

Estrogens regulate oxidative phosphorylation and mitochondrial oxidative stress in the CNS (Gaignard et al., 2018). Furthermore, according to Rettberg et al. (2014), estrogen modulates glucose transport, the glycolytic pathway, the activity of pyruvate dehydrogenase, which is essential in linking glycolysis and the Krebs cycle and other dehydrogenases including succinate and alpha ketoglutarate dehydrogenase, as well as ETC complexes I, III, and IV. Therefore, it seems likely that in females with an LP background, estrogen-modulated bioenergetics (substrate oxidation module) in the brain could protect against detrimental outcomes found in male LP animals, even with the observed reduction in antioxidant capacity.

In regard to the F2 offspring, although several studies have demonstrated an effect of nutritional insult during maternal life in their health, of their F1 offspring, and in the succeeding F2 generation (Reyes-Castro et al., 2015; Saben et al., 2016; Ambeskovic et al., 2017), few reports have evaluated the multigenerational impact of a maternal low-protein diet on parameters related to CVD risk. Harrison and Langley-Evans (2009) deprived pregnant rats of protein (18 vs. 9%) and found that low-protein offspring had higher arterial blood pressure in the following two generations. They also reported that despite an increased blood pressure in the offspring of both sexes, in protein deficient animals, the F1 females had lower arterial blood pressure than the males, corroborating a cardioprotective effect of estrogen in the immediate descendants of protein-deprived females. On the contrary, in the F2 generation, no influence of gender was observed, with blood pressure increased in all LP offspring up to 6 weeks of age. Torrens et al. (2008), using much the same protocol as Harrison and Langley-Evans (2009) also reported that LP offspring in the second generation (at 100 days of age) have higher blood pressure than NP offspring, a result that they associated with endothelial dysfunction.

In the present study, our results demonstrated that F2-LP rats of both sexes responded to a normal laboratory diet in their adulthood by recovering from the nutritional insult suffered by their F1 antecedents. Despite the lower respiratory control in F2 animals of both genders, it is noteworthy that LP mitochondria exhibited increased O_2_ consumption in the ADP-stimulation state, thereby equalizing O_2_ consumption between the NP- and LP- F2 male groups, while allowing higher O_2_ consumption in the females. Moreover, the high O_2_ consumption observed in the uncoupling state suggests an effort by the brainstem mitochondria to deal with oxidative stress (Mailloux and Harper, 2011). It has been shown that uncoupling proteins regulate RS production by modulating the mitochondrial membrane potential (Azzu and Brand, 2010), which might, in turn, result in the increased resistance of CNS neurons to metabolic and oxidative stress through an adaptive shift in energy metabolism (Liu et al., 2006). Furthermore, we show in this investigation that antioxidant capacity is up-regulated in F2-LP progeny, wherein both CAT and GST activity were restored (i.e., F1 vs. F2 pattern), which together with the increase in the redox status, kept oxidative damage in the F2-LP-animals under control. Banos-Gomez et al. (2017) demonstrated that offspring from undernourished grandmothers increased their CAT activity, thereby preventing oxidative damage to the brain. This would suggest an adaptation in the activity of at least one enzymatic antioxidant mechanism that reduces oxidative damage in the second filial generation.

Evidence from the present study suggests that in at least two filial generations following a maternal low-protein insult, brainstem mitochondria may be recovering, or attempting to recover from the detrimental effects programmed by their antecedents by increasing their resilience to peroxides. In regard to possible mechanisms underlying this recovery, Saben et al. (2016) demonstrated that a high-fat/high-sugar diet increases oxidative stress, disrupts mitochondrial function, and alters organellar shape in response to mitochondrial dynamics in the skeletal muscle of mice persisting across three generations, suggesting a possible explanation for the persistence of changes in oxidative parameters that we see in the present report. Moreover, the mitochondrial impairments observed by Saben et al. (2016) were progressively attenuated with each succeeding generation, reinforcing the idea of a gradual recovery/resilience following a one-time occurrence of oxidative damage.

Despite the apparent multigenerational recovery of brainstem mitochondria from an ‘acute’ instance of maternal protein impairment in the F0 generation, however, it appears that F2 offspring in our study remain predisposed to mitochondrial malfunction and oxidative impairments if they themselves experience a low-protein environment during gestation and nursing. This was demonstrated when the re-exposure of second generation animals to the low protein diet (i.e., F2R-LP) increased the production of RS and reduced the O_2_ consumption in the phosphorylated state, regardless of gender. This observation would suggest that during the period of brain development in the F2 generation, a new nutritional insult such as low protein can modify the reparative mitochondrial reprogramming of the brainstem that would normally occur in the second generation post-insult, and result in increased RS and oxidative damage. Furthermore, the damage to F2 generation mitochondria does not appear to be prevented or ameliorated by estrogen, in stark contrast to LP animals of the F1 generation, where a relative resistance to oxidative damage was detected in the female offspring. The higher levels of lipoperoxidation and the lower O_2_ consumption in the mitochondrial uncoupled state in F2-LP females would suggest that the ability of the estrogen to protect mitochondrial function is partially or fully impaired if low-protein is encountered not only in the grandmother’s generation, but in the mother’s generation as well. According to Rodell et al. (2013), mitochondrial function/morphology may fluctuate between maladaptive episodes and adaptive specialization, wherein mitochondrial selection over the course of several generations improves the overall metabolism in an ‘attempt’ to correct the insult that was suffered. The specific mechanisms involved in these effects are unclear, and will need to be investigated in future studies, but those mechanisms could involve epigenetic imprints that can be transferred to the offspring via both nuclear and mitochondrial transcription.

In the present study, since we used male and female from the same nutritional background (i.e., NP or LP) to produce the F2 generation, it remains possible that, the paternal germline could also influence the findings described herein. In this regard (Reyes-Castro et al., 2015) have demonstrated that LP male rats give rise to offspring with different anxiety-related behaviors the appeared to depend on the sex of the progeny. On the other hand, recent, studies have described the possibility of the paternal inheritance of mitochondrial DNA (mtDNA) (Breton and Stewart, 2015; Luo et al., 2018), raising additional possibilities for the inheritance of nutrition-related mitochondrial defects, and challenging the dogma of exclusively matrilineal mtDNA inheritance.

## Conclusion

Our results indicate that a maternal low-protein diet during pregnancy and lactation affects mitochondrial metabolism in the brainstems of F1 progeny of both sexes. However, the response in the male was the overall elevation of oxidative parameters, whereas in the female, both RS and protein oxidation were reduced compared to the control, suggesting an estrogen-dependent protection from nutrition-induced damage in the F1 generation. Moreover, we show that despite the recovery of oxidative balance in the F2 generation of LP rats with normal nutrition in the succeeding generation, the LP-F2 progeny that were ‘re-exposed’ to poor nutrition via their mothers showed marked elevations in RS and in lipid peroxidation. This would suggest that F2 animals with an LP background via their grandmothers remain especially vulnerable to oxidative damage due to later episodes of malnutrition. Results such as these suggest a means by which mitochondrial damage due to malnutrition can span several generations, and leads us to hypothesize a mechanism involving disruption of normal mitochondrial bioenergetics due to impaired ATP synthesis linked to electron coupling-related ADP phosphorylation. Further study will be required, however, to fully investigate and integrate the biochemical parameters that contribute to the transgenerational inheritance of nutrition-dependent mitochondrial damage that eventually leads to CVD.

## Author Contributions

DS conducted the majority of the experiments and analyzed the data. GB and DF conducted several experiments together with DS. TdAS and SS helped with some experiments and take care of the animals. DG and MF helped in the discussion of the experimental design, and with the discussion of the results. DF, BA-d-C, and CL helped with the construction of the experimental design, analyzed the data, discussed the results and manuscript construction.

## Conflict of Interest Statement

The authors declare that the research was conducted in the absence of any commercial or financial relationships that could be construed as a potential conflict of interest.
