# Supplementary figures and images for: Maternal Protein Restriction in Two Successive Generations Impairs Mitochondrial Electron Coupling in the Progeny’s Brainstem of Wistar Rats From Both Sexes
Source: Front Neurosci. 2019 Mar 14;13:203. doi: 10.3389/fnins.2019.00203 (PMC6427765; doi:10.3389/fnins.2019.00203)

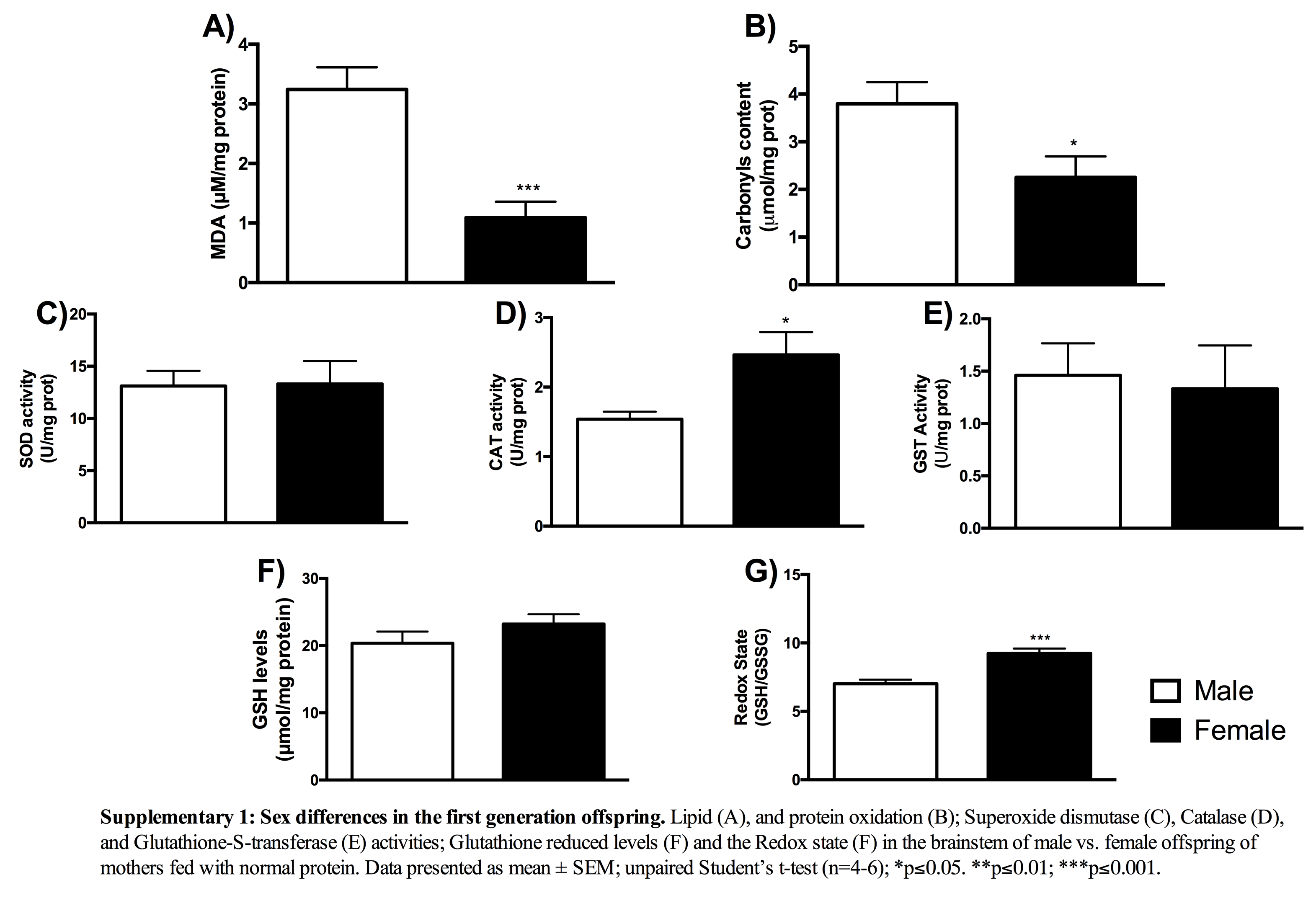

Supplement: Supplementary file 1 [file Image_1.TIFF]
